# RloC: a wobble nucleotide-excising and zinc-responsive bacterial tRNase

**DOI:** 10.1111/j.1365-2958.2008.06387.x

**Published:** 2008-08-07

**Authors:** Elena Davidov, Gabriel Kaufmann

**Affiliations:** Department of Biochemistry, Tel Aviv UniversityRamat Aviv 69978, Israel

## Abstract

The conserved bacterial protein RloC, a distant homologue of the tRNA^Lys^ anticodon nuclease (ACNase) PrrC, is shown here to act as a wobble nucleotide-excising and Zn^++^-responsive tRNase. The more familiar PrrC is silenced by a genetically linked type I DNA restriction-modification (R-M) enzyme, activated by a phage anti-DNA restriction factor and counteracted by phage tRNA repair enzymes. RloC shares PrrC's ABC ATPase motifs and catalytic ACNase triad but features a distinct zinc-hook/coiled-coil insert that renders its ATPase domain similar to Rad50 and related DNA repair proteins. *Geobacillus kaustophilus* RloC expressed in *Escherichia coli* exhibited ACNase activity that differed from PrrC's in substrate preference and ability to excise the wobble nucleotide. The latter specificity could impede reversal by phage tRNA repair enzymes and account perhaps for RloC's more frequent occurrence. Mutagenesis and functional assays confirmed RloC's catalytic triad assignment and implicated its zinc hook in regulating the ACNase function. Unlike PrrC, RloC is rarely linked to a type I R-M system but other genomic attributes suggest their possible interaction *in trans*. As DNA damage alleviates type I DNA restriction, we further propose that these related perturbations prompt RloC to disable translation and thus ward off phage escaping DNA restriction during the recovery from DNA damage.

## Introduction

Bacteria often cope with stress situations by disabling translation (Schneider *et al*., 2003; Hayes and Sauer, 2003; Zhang *et al*., 2005; Wilson and Nierhaus, 2007). The tRNA^Lys^ anticodon nuclease (ACNase) PrrC is a translation disabling device intended to foil phage infection (Amitsur *et al*., 1987; Levitz *et al*., 1990). PrrC is turned on in *Escherichia coli* when the physically associated and genetically linked type Ic DNA restriction-modification (R-M) protein EcoprrI is neutralized by the phage T4-coded peptide Stp (Abdul-Jabbar and Snyder, 1984; Levitz *et al*., 1990; Linder *et al*., 1990; Amitsur *et al*., 1992; Tyndall *et al*., 1994; Penner *et al*., 1995). The resultant cleavage of tRNA^Lys^ 5′ to the wobble base (Amitsur *et al*., 1987) could block T4 late translation (Sirotkin *et al*., 1978). However, the damaged tRNA^Lys^ is normally repaired in consecutive reactions catalysed by T4-coded tRNA ‘healing and sealing’ enzymes. The healing functions are provided by the multifunctional 3′-phosphodiesterase/monoesterase and 5′-polynucleotide kinase protein (Pnk). The healed ends are then sealed by RNA ligase 1 (Rnl1) (David *et al*., 1982; Amitsur *et al*., 1987; Ho and Shuman, 2002). It has been proposed that both proteins evolved to exercise specifically these tRNA repair tasks (Galburt *et al*., 2002; El Omari *et al*., 2006).

PrrC orthologues are distributed among distantly related bacteria, invariably linked to a type Ic R-M system (Amitsur *et al*., 2003; Blanga-Kanfi *et al*., 2006). Those encoded by *Haemophilus influenzae* and *Streptococcus mutans* strains exhibit similar ACNase activities (E. Davidov and S. Blanga-Kanfi, unpubl. results). Therefore, it is conceivable that PrrC's orthologues act in general like the *E. coli* prototype, i.e. disabling translation when an associated DNA restriction function is compromised. Although the ‘second defence line’ provided by PrrC succumbs to phage T4, Stp-encoding but RNA repair-deficient phage (Wietzorrek *et al*., 2006) could in theory be restricted by PrrC.

Ectopic expression of PrrC itself elicits overt ACNase activity (Morad *et al*., 1993) that purifies with an oligomeric form, possibly a PrrC dimer of dimers (Blanga-Kanfi *et al*., 2006; Klaiman *et al*., 2007). PrrC's N-proximal two-thirds constitute an ABC-ATPase domain thought to drive the ACNase activation reaction by hydrolysing GTP in the presence of dTTP (Amitsur *et al*., 2003; Blanga-Kanfi *et al*., 2006). PrrC's remaining third contains a putative catalytic ACNase triad (Blanga-Kanfi *et al*., 2006) as well as residues implicated in tRNA^Lys^ recognition (Meidler *et al*., 1999; Jiang *et al*., 2001; 2002; Blanga-Kanfi *et al*., 2006; Klaiman *et al*., 2007).

**Fig. 1:**
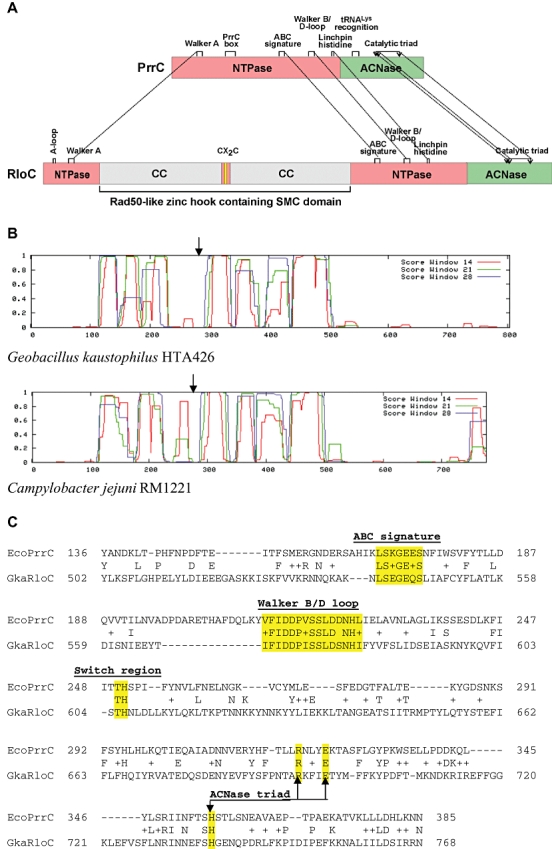
Functional organization of PrrC and RloC. A. Domain alignment. The ATPase domain of PrrC and ATPase head domain of RloC are indicated by pink rectangles, the ACNase domains by green rectangles; predicted α-helical regions flanking the CXXC zinc-hook motif thought to form an antiparallel coiled-coil bundle (CC) (Hopfner *et al*., 2002) are in grey, the gap containing the CXXC motif is in pink and the motif itself is in yellow. Dashed lines connect motifs shared by PrrC and RloC including the Walker A (P-loop), ABC signature, Walker B/D-loop, linchpin histidine/switch region (Moody and Thomas, 2005) and catalytic ACNase triad (Blanga-Kanfi *et al*., 2006). The A-loop [base specificity motif of typical ABC ATPases (Ambudkar *et al*., 2006)] is missing from PrrC whereas the PrrC Box (Blanga-Kanfi *et al*., 2006) and the region implicated in tRNA recognition have been described only in PrrC (Klaiman *et al*., 2007). B. COILED-COIL predictions of RloC orthologues encoded by the indicated bacterial strains. The arrow points at the position of the CXXC motif. C. Alignment of selected *E. coli* PrrC and *G. kaustophilus* HTA426 RloC sequences containing shared functional motifs (highlighted).

RloC, a formerly uncharacterized bacterial protein shares PrrC's putative catalytic ACNase triad and ABC ATPase motifs (Fig. 1). However, RloC's ATPase domain is interrupted by a predicted coiled-coil/zinc-hook insert like that found in the eukaryal/archaeal DNA repair protein Rad50 and the respective bacterial and phage T4 homologues SbcC and gp46 (Hopfner *et al*., 2002; Hopfner and Tainer, 2003). Furthermore, unlike PrrC, RloC only rarely maps to an R-M locus (Miller *et al*., 2005; Table 1). Nonetheless, other genomic attributes discussed later suggest that RloC could interact with an R-M system also *in trans*.

**Table 1:** Genomic attributes of RloC.

Property of RloC orthologues	Frequency
Distribution among microbial genomes	72/850 bacterial; 1/45 archaeal; 0/135 eukaryal
Distribution among bacterial groups	Proteobacteria (53/441), Bacterioidetes (5/36), Firmicutes (8/177), Actinobacteria (5/54), Chlamydiae (0/11), Cyanobacteria (0/37)
Size range	658–897 amino acids
Linkage to a type I or III R-M system	8/73
Presence in strains lacking type I/III R-M systems	10/73
Linkage to cellular DNA metabolism genes	10/73
Linkage to *ardC*^a^	2 (*Rhodobacterales bacterium* HTCC2150; *Bradyrhizobium* sp. BTAi1)
Presence in strains encoding PnkP^b^	2 (*Clostridium thermocellum* ATCC27405, *Bradyrhizobium* sp. BTAi1)
Presence in strains encoding PrrC	2 (*Vibrio splendidus* 12B01, *Brevibacterium linens* BL2)
Strain encoding two different RloC orthologues	1 (*Pseudomonas aeruginosa* UCBPP-PA14)
Presence in possible transposons	10/73

a Anti-DNA restriction factor (Belogurov *et al*., 2000).

b Cellular homologue of T4 Pnk and Rnl 1 (Martins and Shuman, 2005).

We expressed an RloC orthologue encoded by the thermophilic bacillus *Geobacillus kaustophilus* in *E. coli* and began characterizing it *in vivo* and *in vitro*. The recombinant RloC exhibited ACNase activity that differed from PrrC's in (i) substrate preference, (ii) ability to excise the wobble nucleotide and (iii) susceptibility to zinc ions. Mutational data confirmed RloC's catalytic triad assignment and implicated its zinc-hook motif in regulating the ACNase function. These conclusions, taken with the well-documented alleviation of type I DNA restriction after DNA damage (Blakely and Murray, 2006), led us to propose that RloC responds to DNA damage and/or the consequent alteration of the associated R-M enzyme by disabling translation. This, in turn, could ward off phage that might escape DNA restriction during the recovery from DNA damage. Moreover, the excision of the wobble nucleotide could encumber the restoration of the damaged tRNA by phage tRNA repair enzymes and thus render RloC a more powerful antiviral device than PrrC.

## Results

### RloC – a distant PrrC homologue with a Rad50-like N-domain

A blast search (Altschul *et al*., 1997) using *E. coli* PrrC as a query revealed besides two dozen orthologues (Amitsur *et al*., 2003; Blanga-Kanfi *et al*., 2006) a threefold more abundant group of distant homologues that share PrrC's ABC ATPase motifs (Fig. 1; Table 1; Table S1). Manual adjustment identified in their C-proximal portions a conserved Arg–X_3_–Glu–X_36−52_His motif (where Ser often precedes His) reminiscent of PrrC's putative catalytic ACNase triad (Arg^320^–Glu^324^–His^356^) (Fig. 1B). Genes encoding such proteins have been detected as inserts within type I DNA R-M loci of various *Campylobacter jejuni* strains and named accordingly *rloC*, *rloE* or *rloG* (restriction linked orf; Miller *et al*., 2005). Yet, of the 72 specimens found in the sequenced bacterial genomes of the NCBI database only eight mapped to a type I or the related type III R-M locus and 12 existed in bacteria lacking either (Table S1). Nonetheless, the entire group is referred here to as RloC.

The RloC orthologues are about twice the size of PrrC's (658–897 versus 341–416 aa respectively). This increase owes mainly to an insert that splits the ABC ATPase domain of RloC (Fig. 1A), similar to the inserts found in the eukaryal/archaeal DNA repair protein Rad50 and the homologous bacterial SbcC and phage T4 gp46 (Hopfner *et al*., 2002). Such inserts comprise a largely α-helical region interrupted by a central loop containing the conserved CX_2_C sequence. The CX_2_C motif is named ‘zinc-hook’ because its cysteines co-ordinate Zn^++^ at a dimerization interface. The flanking, α-helical portions fold back into an antiparallel coiled-coil bundle that emerges from the ATPase head domain. Two such protrusions linked through their apical zinc hooks bridge two DNA molecules or segments tethered to the ATPase head domains via an associated DNase (Hopfner *et al*., 2002). RloC's insert could form a similar zinc-hook/coiled-coil protrusion based on its predicted secondary structure (by Jpred, Cuff *et al*., 1998; Fig. S1) and coiled-coil content (Lupas *et al.*, 1991; Fig. 1B). However, Rad50, SbcC and T4 gp46 lack an equivalent of RloC's C-region that shares the putative catalytic ACNase triad and predicted secondary structure of PrrC's.

Several other attributes of RloC are noteworthy. First, the sequences preceding the RloC orfs, their rare codon usage and nature of initiation codons (13 GUG and one UUG among 70 full-fledged RloC orfs) predict a low expression level. Second, two RloC orthologues are linked to the anti-DNA restriction factor ArdC (Belogurov *et al*., 2000), hinting that at least they could be mobilized in ‘PrrC's way', i.e. through inactivation of an associated DNA restriction nuclease. Third, some RloC orthologues exist in bacteria encoding also (i) another RloC orthologue, (ii) PrrC or (iii) a composite cellular homologue of the T4 tRNA healing and sealing enzymes termed PnkP (Martins and Shuman, 2005). The motifs RloC shares with PrrC, its unique features and diverse genomic attributes (Table 1; Table S1) hinted that RloC could also function as a regulated, translation-disabling ACNase but not necessarily in the same situations in which PrrC is mobilized. We began addressing these assumptions by investigating an RloC orthologue from the thermophilic bacillus-related strain *G. kaustophilus* HTA426 (Takami *et al*., 2004).

### Expression of *G. kaustophilus* RloC in *E. coli* elicits ACNase activity

**Fig. 2:**
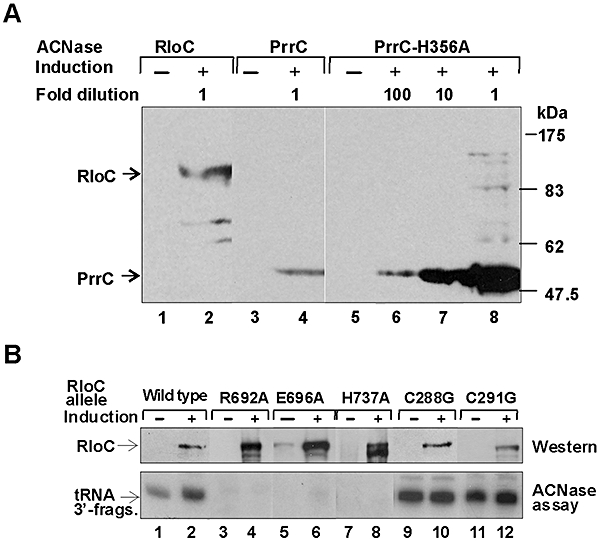
Expression of wild-type and mutated *G. kaustophilus* RloC forms in *E. coli*. A. RloC's expression is limited. *E. coli* Rosetta encoding wild-type *G. kaustophilus* RloC (lanes 1, 2), wild-type PrrC (lanes 3, 4) or the inactive PrrC-H356A mutant (lanes 5–8) not induced (odd lanes) or induced with 100 μM IPTG (even lanes) were lysed, the cellular proteins separated by SDS-PAGE and the recombinant ACNase proteins monitored by immunoblotting using an anti-His_6_ monoclonal antibody. The extract with the inactive PrrC mutant H356A was diluted in lanes 6 and 7 100- or 10-fold respectively. B. *In vivo* ACNase activity and protein level of RloC catalytic triad and zinc-hook mutants. *E. coli* Rosetta cells encoding the indicated RloC alleles were analysed for RloC protein level and *in vivo* ACNase activity as detailed in *Experimental procedures*.

**Fig. 3:**
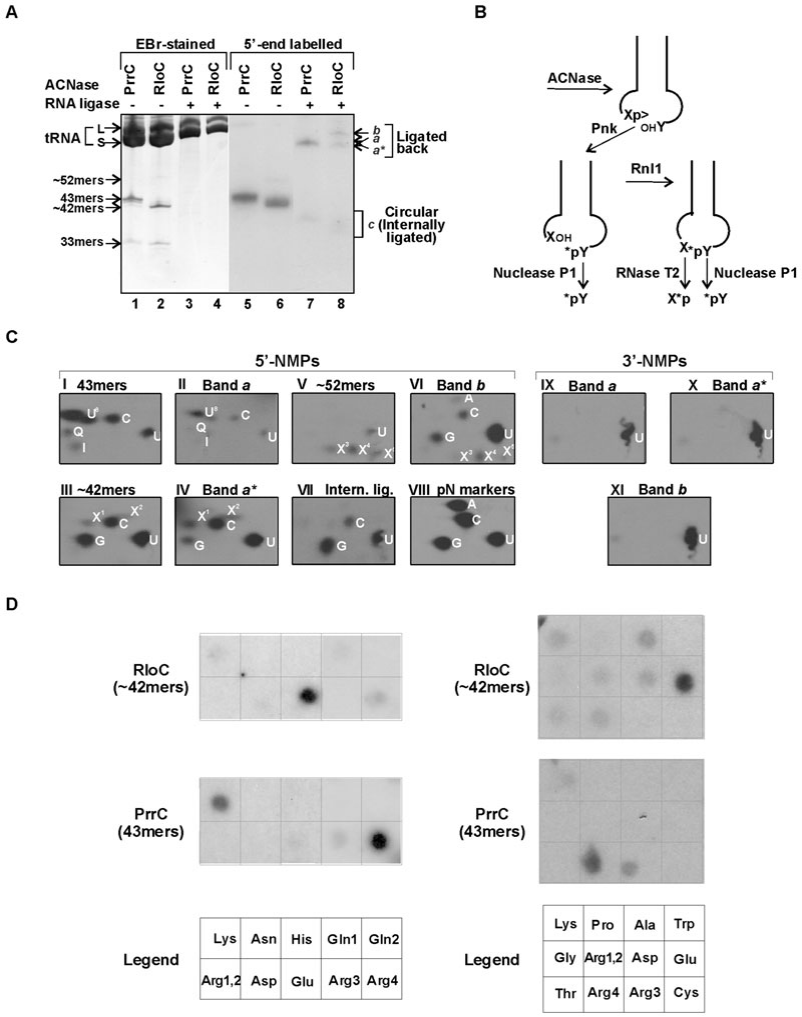
RloC-expressing cells manifest ACNase activity. A. Total RNA samples isolated from cells expressing PrrC (odd lanes) or RloC (even lanes) were separated by denaturing polyacrylamide gel electrophoresis as such (lanes 1, 2) or after being 5′-end-labelled by T4 Pnk (lanes 5, 6). The non-labelled RNA fractions were also further incubated with T4 Pnk and Rnl 1 (lanes 3, 4) and then ligated with Rnl1 (lanes 7, 8). The gel was then stained with ethidium bromide (lanes 1–4) or autoradiographed (lanes 5–8). 33mers are 5′-cleavage products generated by either ACNase. 43mers are 3′-cleavage products generated by PrrC, ∼42mers and ∼52mers 3′-cleavage products generated by RloC. Band *a* contains the ligated PrrC cleavage products, bands a* and *b* contain the RloC counterparts, and bands *c* represents presumable internally ligated (circular) cleavage products of either ACNase. B. Scheme describing the cleavage of the ACNase substrate, 5′-end-labelling of the 3′-cleavage product, the subsequent ligation and the release of the labelled nucleotides from the labelled fragments or ligated-back molecules by the indicated nucleases. C. 2D TLC of radiolabelled nucleotides released by nuclease P1 (panels I–VII) or RNase T2 (panels IX–XI) from the indicated labelled RNA preparations. The 5′-end-labelled 43mers, ∼42mers and ∼52mers (Fig. 4A, lanes 1, 2), their ligated-back derivatives of bands a, a* and *b* (lanes 3, 4) and the circularized forms of RloC's products (lane 3) were digested by nuclease P1 and the released radiolabelled nucleotides separated by 2D TLC, as indicated. 5′-NMP markers are shown in panel VIII. The 3′-NMPs released from the indicated ligated-back derivatives by RNase T2 were similarly separated. The identity of U^8^ (panels I, II) was ascertained by subsequent separation on PEI-cellulose TLC (not shown). X^1^–X^5^ indicate apparent modified or hypomodified nucleotides that were not identified. D. Identification of tRNA species cleaved by PrrC or RloC. The 5′-end-labelled 43mers generated by PrrC (Fig. 4A, lane 1) or RloC's ∼42mers (Fig. 4A, lane 2) were hybridized to dot blots containing antisense DNA oligonucleotides corresponding to the indicated *E. coli* tRNA species described by Jiang *et al*. (2001) and in Table S2.

Attempted overexpression of *G. kaustophilus* RloC in *E. coli* resulted in a minuscule yield of the recombinant protein, comparable to that of wild-type PrrC but ∼100-fold lower than an inactive PrrC mutant, as inferred from the relative intensities of the respective His_6_-tag immunoblotting signals (Fig. 2A, compare lane 2 with 4, 6 and 8). RloC's expression limited not only its own production but also cell growth (Fig. S2, line C). These limitations resembled those seen with PrrC's overexpression (Meidler *et al*., 1999; Blanga-Kanfi *et al*., 2006) (Fig. S2, line A) and, hence, could reflect the anticipated ACNase activity. As shown in Fig. 3A, such activity was detected by staining the tRNA cleavage products formed in the RloC expressing cells (lane 2) or by *in vitro* labelling their 5′-OH cleavage termini (lane 6), as done to visualize PrrC's *in vivo* products (lanes 1, 5). In this *in vivo* assay, RNA fractions isolated from PrrC or RloC expressing cells were separated by denaturing gel electrophoresis, as such (lanes 1, 2) or after being 5′-end-labelled using T4 Pnk (lanes 5, 6). Staining the untreated RNA with ethidium bromide revealed the expected ∼33 and ∼43 nt cleavage products of PrrC that match in size tRNA^Lys^ residues 1–33 and 34–76 respectively (lane 1). It should be noted that overexpression of PrrC causes cleavage also of secondary substrates, mostly with anticodons resembling that of tRNA^Lys^ (Meidler *et al*., 1999). Some of the secondary substrates differ from tRNA^Lys^ in the number of D-loop nucleotides and therefore yield ‘33mers’ 1 nt shorter or longer. Nonetheless, for convenience we refer to all 5′-fragments generated by PrrC as 33mers. The RloC-expressing cells (lane 2) contained a pair of major product bands. One of them coincided with PrrC's 33mers. The other travelled somewhat faster than PrrC's 43mers, as if comprising chains 1 nt shorter (termed ∼42mers). A minor band estimated to contain ∼52mers was also detected in the RloC lane. As expected, the PrrC's 43mers were 5′-end-labelled (Fig. 3A, lane 5) and so were RloC's ∼42 and ∼52mers (lane 6), indicating that the latter two also constituted 3′-cleavage products.

Incubating the unfractionated RNA samples with T4 Pnk and Rnl1 caused the disappearance of the stained tRNA fragments (lanes 3, 4), possibly due to their reunion. In agreement, RNA ligase treatment shifted a sizeable fraction of the 5′-end label into the tRNA size range (lanes 7, 8). The remaining label was converted into faster-migrating derivatives, presumably circular, intramolecularly ligated products (designated *c*). The tRNA-sized, ligated-back cleavage products of PrrC clustered in a single band migrating with tRNA species of ∼76 nt (marked with S), probably containing restored PrrC substrates (lane 7, designated *a*). The ligated-back RloC products (lane 8) were distributed between two fractions of comparable intensity. One, designated a*, migrated slightly ahead of PrrC's counterparts of band *a*, as if 1 nt shorter. The other, designated *b*, migrated like long tRNA species (marked with L). Thus, RloC's expression elicited ACNase activity that could differ from PrrC in substrate and/or cleavage site specificity. Below we refer to this activity as RloC ACNase or RloC as data shown later suggested that it resided in the RloC protein.

### RloC ACNase differs from PrrC in cleavage site specificity and substrate preference

The relation between the major and minor RloC cleavage products and the derived ligation products of bands a* and *b* was investigated by comparing the 5′-end groups of the former with the labelled nucleotides incorporated in the latter. These entities were released by digesting the RNA fractions with the non-specific nuclease P1 and separated by two-dimensional thin-layer chromatography (2D TLC) (Fig. 3B and C). PrrC's 43mers and the ligated-back derivatives of band *a* were similarly analysed. As expected, the main labelled nucleotide released from the latter two was 5-methylaminomethyl-2-thiouridine 5′-phosphate (pU^8^) (Fig. 3C, panels I, II), i.e. the wobble nucleotide of PrrC's major substrates. Three minor components (C, Q, I) could represent other wobble nucleotides derived from less reactive PrrC substrates. However, the release of labelled U was puzzling as *E. coli* tRNAs do not feature unmodified U at the wobble position. We suspect that the liberated U originated from hypomodified PrrC substrates that could accumulate following the disruption of mature species and consequent enhancement of tRNA transcription. A relevant observation is the accumulation of hypomodified tRNA^Lys^ forms lacking anticodon loop modifications during the attempted overexpression of this species (Commans *et al*., 1998).

The RloC counterparts released different sets of labelled products. The most conspicuous of them were the following. The ∼42mers yielded comparable amounts of pG, pC and pU and traces of modified or hypomodified nucleotides termed pX^1&2^ (panel III). The ∼52mers released similar amounts of modified or hypomodified nucleotides termed pX^3,4&5^ as well as pU (panel V). The ligated-back band a* derivatives yielded mainly pC and pU and less of pG and pX^1&2^ (panel IV) while the band *b* derivatives released mainly pU and less of pC, pG and pX^3,4&5^ (panel VI). Table 2 lists these end groups.

**Table 2:** Nucleotides at cleavage termini generated by PrrC or RloC.

RNA fraction	5′-NMPs	3′-NMPs
43mers	U^8^ ≫ C = U > Q = I	
Band *a*	U^8^ ≫ C = U > Q = I	U
∼42mers	G > C = U ≫ X^1^ = X^2^	
∼52mers	U = X^3^ = X^4^ = X^5^	
Band a*	C ≫ U = G = X^3^ = X^4^ = X^5^	U
Band *b*	U ≫ C = G = X^3^ = X^4^ = X^5^	U
Band *c*	G ≫ U = C	

**Fig. 4:**
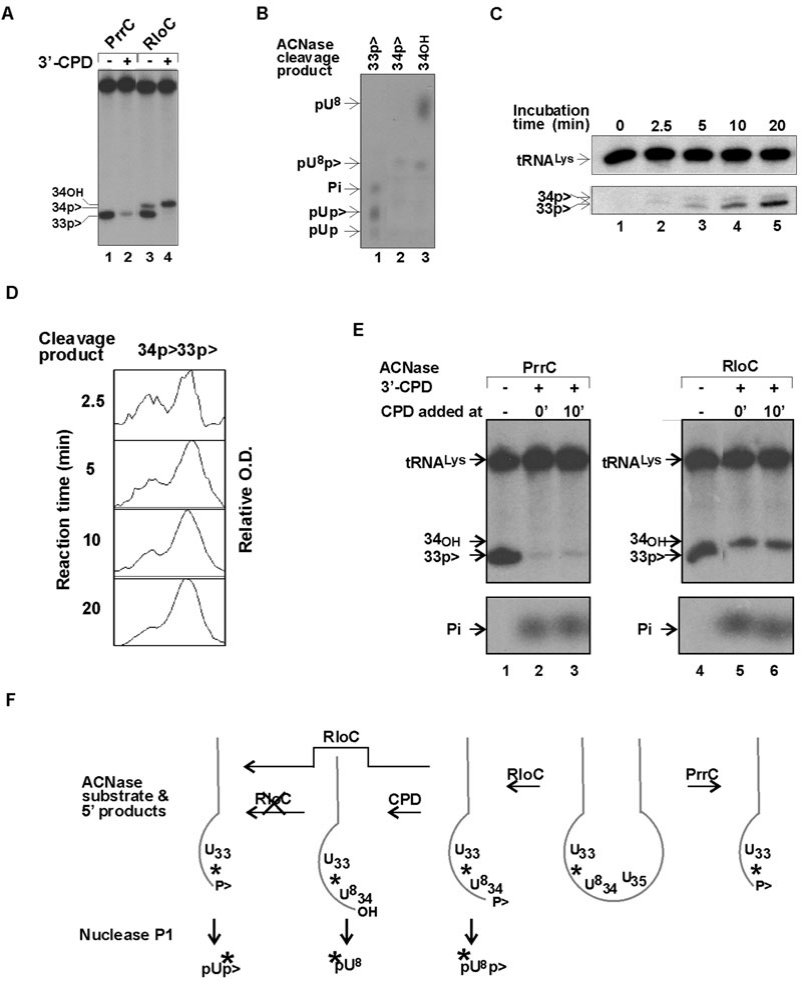
*In vitro* cleavage of tRNA^Lys^ by PrrC or RloC. A. Cleavage products of tRNA^Lys^ generated by PrrC or RloC. The tRNA^Lys^ substrate labelled at the 33p34 junction was incubated with PrrC (lanes 1, 2) or RloC (lanes 3, 4) alone (lanes 1, 3) or in the presence of T4 Pnk providing 3′-cyclic phosphodiesterase/monoesterase (CPD) activities (lanes 2, 4). The products were separated by denaturing gel electrophoresis as detailed in *Experimental procedures*. B. 3′-end analysis of 33p>, 34p> and 34_OH_. The indicated labelled products obtained by tRNA^Lys^ digestion with RloC (A, lane 1) or RloC and T4 Pnk (lane 2) were further digested with nuclease P1 and separated by PEI-thin-layer chromatography. The digestion products were identified by their position relative to markers produced by digesting tRNA^Lys^ labelled at the 33p34 junction with (i) nuclease P1 to yield labelled pU^8^, (ii) RNase T2 to yield labelled Up, (iii) PrrC followed by nuclease P1 to yield labelled pUp>. The identity of pU^8^p> derived from 34p> by nuclease P1 digestion was ascertained by the ability to dephosphorylate it into labelled pU^8^ by incubation with T4 Pnk. C. Time-course of tRNA^Lys^ cleavage by RloC. D. Proportions of the 34p> and 33p> cleavage products of tRNA^Lys^ generated during the incubation with RloC. Shown are scanned profiles of the regions containing 34p> and 33p> in the indicated lanes of (C). E. Constancy of the 34p> fraction accessible to CPD. The cleavage of tRNA^Lys^ by RloC was performed in the presence of CPD added either at the onset of the RloC reaction with RloC or 10 min later, as indicated. F. Scheme of tRNA^Lys^ cleavage by PrrC or RloC and subsequent analyses of the cleavage products. The asterisk indicates the radiolabelled phosphate at the PrrC cleavage junction. The original substrate is represented by its anticodon stem loop region with positions of the canonical U_33_, the wobble base 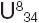 and second anticodon base U_35_ indicated. RloC cleaves the substrate initially 5′ to U_35_ yielding a 5′-fragment containing the radiolabel at the internal 33p34 position. The second cleavage by RloC at PrrC's site exposes the label to the phosphodiesterase/monoesterase activities of T4 Pnk (CPD) but can be pre-empted by prior 3′-dephosphorylation of 34p>. Nuclease P1 releases the indicated end groups from the various labelled products generated by RloC with or without CPD.

The indicated labelled RNA products of Fig. 4A were digested by nuclease P1 or RNases T2. The respectively labelled 5′-NMPs and 3′-NMPs released by these digestions were identified by 2D TLC (Fig. 4B) as described in the legend to Fig. 4. The relative amounts of the 5′-NMPs are described as follows: ≫, much greater; >, greater; =, comparable.

It follows from the end group data that the RloC ∼42mers with 5′-C and part of those with 5′-U were efficiently converted into the short tRNA-like molecules of band a* (compare panels III and IV), possibly by joining onto the cognate 5′-tRNA fragments. In contrast, the incorporation of 5′-G ∼42mers into tRNA-like molecules, whether of band a* or *b* (compare panel III with IV and VI), was far less efficient. Instead, the ∼42mers with 5′-G seemed to have largely undergone intramolecular ligation (panel VII). Less clear was why a sizeable fraction of the ∼42mers was converted into the longer band *b* molecules (compare panels III and VI). One possibility considered is that RloC generated these ∼42mers by cleaving precursors of short tRNA species carrying 5′ leader sequences. Alternatively, they were inadvertently ligated to non-specific RNA fragments. Less likely seems that they originated from long species cleaved at the variable arm as tRNA modifying/processing proteins usually act in a site-specific manner. However, the ∼52mers converted into band *b* products could originate by ‘legitimate’ ACNase cleavage of long species (compare panels V and VI) or derived from precursors of short tRNA species carrying 3′ tails.

The nature of the 5′-cleavage termini generated by RloC indicated that it differed from PrrC both in cleavage site specificity and in substrate preference. The first conclusion was drawn from the dearth of typically modified wobble nucleotides at the 5′-end of RloC's ∼42mers and abundance of unmodified U at this position (Fig. 3C, panel III) although unmodified U is not an *E. coli* wobble base. These facts, taken with the apparent 1 nt difference between the 3′-fragments generated by the two ACNases (Fig. 3A, lanes 1, 2), suggested that RloC cleaved its substrates 1 nt downstream to PrrC's site. On the other hand, the similar size of the 5′-fragments and 1 nt size difference between the ligated-back molecules of bands *a* and a* hinted that RloC cleaved its substrates also at PrrC's site.

The distinct substrate preferences of RloC was deduced from the abundance of G at the 5′-ends of its ∼42mers (Fig. 3C, panel III) as opposed to the absence of G from the anticodons of the major substrates cleaved by PrrC (tRNA^Arg3,4^, tRNA^Lys^; Fig. 4D). Hence, RloC could cleave also tRNA species not recognized by PrrC. The substrate preferences of RloC and PrrC were more closely evaluated by hybridizing their 5′-labelled cleavage products to dot blots containing antisense oligonucleotides representing various *E. coli* tRNA species (Fig. 3D). The oligonucleotide probes in the left panels represented mainly species cleaved by overexpressed PrrC (Meidler *et al*., 1999). Among them PrrC's 43mers lighted up predominantly the tRNA^Arg4^ (U^8^CU anticodon) dot, a rare *E. coli* species over-represented in the strain used here to express the ACNases. PrrC's 43mers hybridized somewhat less efficiently to the tRNA^Lys^ (U^8^UU) probe and to a lesser or similar extent to the tRNA^Arg3^ (U^8^CU) and tRNA^Glu^ (U^8^UC) probes. The relative paucity of the tRNA^Lys^ fragments has been previously accounted for by PrrC's overexpression (Meidler *et al*., 1999). In contrast, RloC's ∼42mers interacted predominantly with the tRNA^Glu^ dot and to lesser extents with those of tRNA^Lys^, tRNA^Arg4^ and tRNA^Gln1^ (CUG). The right panel contained also probes representing tRNA species with G or C as the second anticodon letter as the end group analysis showed that RloC cleaved also such species (Fig. 4C, panel III). Indeed, RloC's ∼42mers but not PrrC's 43mers lighted up tRNA^Thr^, tRNA^Ala^ (G as the second letter), tRNA^Arg1,2^ and tRNA^Gly^ (C as the second letter) dots similar to the tRNA^Lys^ and tRNA^Arg4^ dots but less than that of tRNA^Glu^.

### RloC excises the wobble nucleotide

As mentioned, the 3′-tRNA fragments produced by the two ACNases differed in size by 1 nt and so were the ligated-back derivatives of bands *a* and a* whereas the sizes of the 5′-tRNA fragments seemed identical (Fig. 3A). These facts hinted that RloC cleaved its substrates both one position 3′ to and at PrrC's site, excising the wobble nucleotide in the process. This assumption was examined by determining the 3′-cleavage termini generated by each ACNase. To this end, the ligated-back molecules of bands a, a* or *b* were digested with the non-specific RNase T2 (Fig. 3B) and the liberated radioactive 3′-NMPs separated by 2D TLC (Fig. 3C, panels IX-XI). As shown, in each case only labelled Up was liberated. With PrrC, which cleaves its substrates 3′ to the canonical U_33_, this result was anticipated. However, to generate such 3′-termini RloC had to cleave its substrates not only 3′ to but also at PrrC's site. This conclusion was reinforced by the absence of the wobble nucleotide from the ligated-back tRNA^Lys^ fragments generated by RloC (Fig. S3) and RloC's *in vitro* cleavage specificity described next (Fig. 4).

The cleavage site specificities of PrrC and RloC were compared *in vitro* using their crude S30 or IMAC-purified fractions and a tRNA^Lys^ substrate radiolabelled at the 33p34 cleavage junction (Amitsur *et al*., 1989). The choice of this substrate seemed justified because tRNA^Lys^ was among the substrates cleaved by RloC *in vivo* (Fig. 3D) and the particular labelling mode facilitated the intended comparison. As expected, PrrC converted this substrate into tRNA^Lys^ fragment 1–33 labelled at the 3′-cyclic phosphate (designated 33p>, Fig. 4A, lane 1) and this label was readily removed by the 3′-phosphodiesterase/monoesterase activities (CPD) of T4 Pnk (Fig. 4A, lane 2, Fig. 4E, lanes 2, 3). In contrast, RloC converted the tRNA^Lys^ substrate into two labelled products. One coincided with 33p> the other was slightly retarded and termed 34p> (Fig. 4A, lane 3). Including CPD in the RloC reaction mixture abolished the 33p> and 34p> bands and yielded instead a yet slower migrating labelled product termed 34_OH_ (lane 4). Digesting 33p>, 34p> or 34_OH_ with nuclease P1 converted their label in respective order into pUp> (Fig. 4B, lane 1), pU^8^p> (lane 2) or pU^8^ (lane 3). These results indicated that RloC cleaved tRNA^Lys^ both 1 nt 3′ to and at PrrC's site, yielding in respective order 34p> and 33p>. During the RloC reaction the proportion of 34p> decreased while 33p> accumulated (Fig. 4C and D), suggesting that RloC performed the two cleavages successively, yielding first 34p> and then 33p>. Accordingly, CPD prevented the second cleavage by converting 34p> into 34_OH_. These interpretations are depicted in Fig. 4F. However, only a minor fraction of 34p> was accessible to CPD. This was inferred from the small amount of 34_OH_ formed in the presence of CPD compared with that of labelled Pi liberated from 33p> by CPD (Fig. 4E, lane 5, 6) or of 33p> accrued in the absence of CPD (lane 4). Moreover, the level of Pi liberated from 33p> was not significantly changed by the later addition of CPD to the RloC reaction mixture (lanes 5, 6) or increasing the CPD/RloC ratio (not shown). These results suggested that RloC performed its successive cleavages essentially in a processive manner, occluding the bulk of the 34p> intermediate. Presumably, the accessible fraction of 34p> represented molecules inadvertently dissociated from the unstable ACNase under the *in vitro* conditions.

### Mutating RloC's catalytic triad abolishes its ACNase activity

The putative catalytic ACNase triad of *E. coli* PrrC: Arg^320^, Glu^324^ and His^356^ (Blanga-Kanfi *et al*., 2006) is matched in *G. kaustophilus* RloC by Arg^692^, Glu^696^ and His^737^ (Fig. 1C). Replacing any of these RloC residues by Ala abolished the *in vivo* ACNase activity, increased the RloC protein level ∼10-fold (Fig. 2B, lanes 4, 6, 8) and abolished its toxicity (Fig. S3, lines C–E). These data suggested that RloC itself harbours the ACNase function and confirmed its anticipated translation disabling ability and consequent self-limiting expression. They also supported RloC's catalytic triad assignment and reinforced PrrC's.

### RloC and ACNase co-purify

**Fig. 5:**
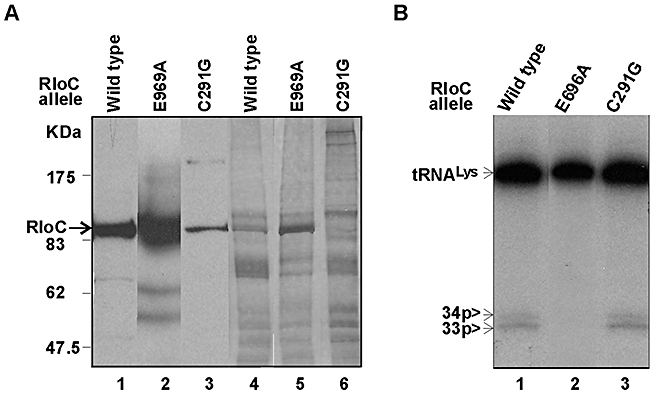
RloC and ACNase co-purify. A. Samples of the IMAC-purified wild-type RloC (lanes 1, 4), the ACNase-null E696A mutant (lanes 2, 5) or the zinc-hook mutant C291G (lanes 3, 6) derived from the same amount of total cell protein were separated by SDS-PAGE and monitored by immunoblotting using anti-His_6_-tag antibodies (lanes 1–3) or by silver staining (lanes 4–6). kDa, protein size markers. B. Samples of the indicated RloC alleles like those described in (A) were assayed for tRNA^Lys^ cleavage activity as in Fig. 4.

RloC eluted from the IMAC column retained the specific tRNA^Lys^ cleaving activity (Fig. 5A, lanes 1, 4; Fig. 5B, lane 1). In contrast, no ACNase activity was detected with the purified E696A mutant despite its much higher protein level (Fig. 5A, lanes 2, 5; Fig. 5B, lane 2). Wild-type RloC was readily detected by Western blotting. Despite its minuscule yield, it could also be distinguished from co-eluting non-specific proteins by staining. Namely, its stained band coincided with the conspicuous band of the abundant E696A mutant protein. Moreover, it stuck out over the background provided by the zinc-hook mutant C291G. The latter mutant, further discussed below, was far more active than wild-type RloC and, consequently, expressed at a yet lower yield (Fig. 5A, lanes 3, 6; Fig. 5B, lane 3).

**Fig. 6:**
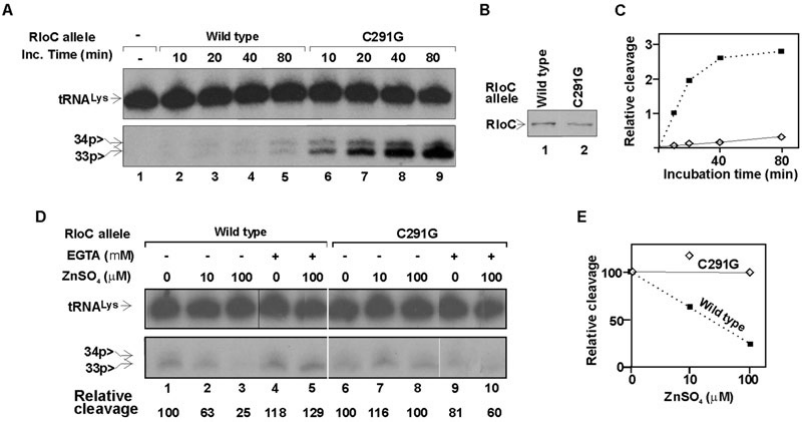
Effects of Zn^++^ and EGTA on wild-type RloC or its zinc-hook mutant. A. Time-course of tRNA^Lys^ cleavage by wild-type RloC or the zinc-hook mutant C291G. B. Western analysis of the S30 aliquots of the two RloC forms assayed in (A). C. Relative tRNA^Lys^ cleavage versus incubation time with the indicated RloC allele. D. Effect of Zn^++^ and EGTA on the ACNase activity of the indicated RloC alleles. IMAC-purified fractions of the indicated RloC alleles were used in this assay. The reaction was performed under the standard conditions described in *Experimental procedures* and the incubation time was 30 min. E. Relative tRNA^Lys^ cleavage activity versus zinc sulphate concentration.

The co-elution of wild-type RloC and ACNase activity and failure to detect it in the purified E696A mutant fraction suggested that RloC itself rather than a non-specific *E. coli* protein harboured this activity. An alternative possibility that the observed ACNase activity was conferred by an *E. coli* ACNase that specifically associated with RloC was disfavoured because (i) the *E. coli* host used is not known to encode such an entity, (ii) the RloC orthologue investigated originated from a distantly related bacterium, and (iii) the activity was abolished by mutating any residue of RloC's putative catalytic ACNase triad and was enhanced by mutating its zinc-hook cysteines (Figs 2B, 5 and 6).

### Zinc-hook mutations enhance and Zn^*++*^ inhibits the RloC ACNase

The zinc-hook mutations C288G or C291G dramatically enhanced RloC's *in vivo* ACNase activity. This was inferred from the massive accumulation of the ACNase reaction products even without inducing the expression of the mutant proteins (Fig. 2B, lanes 9, 11) and the exacerbated cytotoxicity of these mutants (Fig. S2, lines F, G). The C291G mutant was compared with wild-type RloC also *in vitro.* When crude S30 fractions containing similar amounts of the two forms were assayed, the mutant cleaved tRNA^Lys^ ∼20-fold faster than the wild-type protein (Fig. 6A and C). The response of the wild-type and C291G forms to Zn^++^ was determined by assaying their IMAC-purified fractions from which endogenous Zn^++^ was largely removed. Aliquots of comparable activity were used in this case. Adding zinc sulphate to the reaction mixture inhibited tRNA^Lys^ cleavage by wild-type RloC in a dose-dependent manner, up to a fourfold at the physiological Zn^++^ level of 0.1 mM (Fig. 6D, lanes 1–3). Moreover, adding 1 mM of the zinc chelator EGTA, alone or in the presence of 0.1 mM zinc sulphate, slightly stimulated the wild-type activity (lanes 4, 5). In contrast, zinc sulphate did not affect the mutant's activity (lanes 6–8) and EGTA inhibited it (lanes 9, 10). These data suggested that RloC's zinc-hook interaction with Zn^++^ downregulated the ACNase function.

## Discussion

### RloC is a novel ACNase

Overexpression of *G. kaustophilus* RloC in *E. coli* disrupted the anticodon loops of multiple tRNA species. The favoured substrate of this ACNase under the experimental conditions employed appeared to be tRNA^Glu^ (Fig. 3D). However, the identity of its natural substrate remains unknown due to the following reasons. First, overexpression of PrrC elicits cleavage not only of the natural substrate tRNA^Lys^ (Amitsur *et al*., 1987) but also of other species, mostly with similar anticodons (Meidler *et al*., 1999). Hence, it is possible that RloC's overexpression also resulted in cleavage of ‘unintended’ substrates. Second, to compensate for the rare codons of the ACNase orfs we used an *E. coli* Rosetta strain containing the plasmid pRARE, which overexpresses tRNAs that decode codons rarely used by *E. coli* including Arg (AGG, AGA), Leu (CUA), Pro (CCC) and Ile (AUA) codons. The pRARE insert encoding these species also encodes Thr, Met and Tyr tRNA species. As mentioned, one of these rare species (tRNA^Arg4^) was the major substrate of the overexpressed PrrC, yielding far more cleavage products than the natural substrate tRNA^Lys^ (Fig. 3D). Third, we assume that the depletion of susceptible tRNA species by the ACNase could enhance tRNA transcription and, in turn, lead to the accumulation of incompletely processed intermediates (Commans *et al*., 1998), some of which could also be cleaved. Fourth, the natural substrate of *G. kaustophilus* RloC could be absent or under-represented into the *E. coli* host while other species absent from *G. kaustophilus* could be accidentally cleaved by the heterologous ACNase. These problems precluded the identification of the natural substrate(s) of RloC but not comparing its biochemical properties with PrrC's under similar experimental conditions.

Several observations indicated that RloC itself harboured this ACNase activity rather than an indigenous *E. coli* protein induced or activated by RloC. First, mutating RloC's putative equivalents of PrrC's catalytic ACNase triad, Arg^692^, Glu^696^ or His^737^, abolished in each case RloC's *in vivo* ACNase activity (Fig. 2B). Second, IMAC-purified wild-type RloC but not the E696A mutant exhibited the specific ACNase activity characterized by the appearance of both 34p> and 33p> (Fig. 5B). Third, Zn^++^ inhibited the ACNase activity of wild-type RloC but not the zinc-hook mutant C291G (Fig. 6). Fourth, RloC's zinc-hook mutant C291G was far more active as an ACNase than wild-type RloC both *in vivo* (Fig. 2B) and *in vitro* (Fig. 6). An alternative interpretation that the observed ACNase activity was conferred by a protein associated with RloC could be invoked by analogy with the DNases associated with Rad50 (Mre11) or SbcC (SbcD) (Cromie *et al*., 2001; Connelly and Leach, 2002). However, even if an indigenous *E. coli* ACNase existed, it seems improbable that it would specifically associate with the heterologous *G. kaustophilus* RloC and that such association will be disrupted by mutating any of RloC's catalytic ACNase triad residues or enhanced by RloC's zinc-hook mutations. Furthermore, as mentioned, Rad50 and SbcC lack an equivalent of RloC's C-region that shares with PrrC's C-domain the catalytic ACNase triad and predicted secondary structure. As later explained, a more likely candidate for tethering RloC to DNA could be an associated DNA R-M enzyme.

### A zinc-hook switch could regulate RloC ACNase

Translation-disabling tools are potentially harmful to their host and therefore must be kept inactive, ready to be unleashed when required. For example, PrrC ACNase is silenced in uninfected *E. coli* by the associated DNA restriction endonuclease EcoprrI (Abdul-Jabbar and Snyder, 1984; Levitz *et al*., 1990; Linder *et al*., 1990; Amitsur *et al*., 1992; Tyndall *et al*., 1994) and activated by Stp, a co-opted phage T4-coded inhibitor of EcoprrI (Penner *et al*., 1995). RloC ACNase could be as harmful and therefore also mobilized only in specific dire situations. The overt ACNase activity of RloC observed here need not contradict the anticipated silencing as this activity was manifested by a recombinant RloC form overexpressed in a heterologous host. These conditions could (i) amplify a weak basal activity, (ii) overwhelm a cognate silencing entity, if present in the heterologous cell, and/or (iii) yield partially degraded and activated forms akin to the hyperactive zinc-hook mutants. Conversely, RloC's latency and timely activation in its natural milieu could be safeguarded by a (i) low expression level, (ii) cognate silencing partner(s), and/or (iii) regulatory switch responsive to specific signals, e.g. RloC's zinc hook. The latter possibility was inferred from the dramatic stimulation of RloC's ACNase activity by zinc-hook mutations (Fig. 2B) and sensitivity of the wild type but not zinc-hook mutant activity to inhibition by Zn^++^ (Fig. 6).

### DNA damage and DNA restriction alleviation as possible triggers of RloC ACNase

**Fig. 7:**
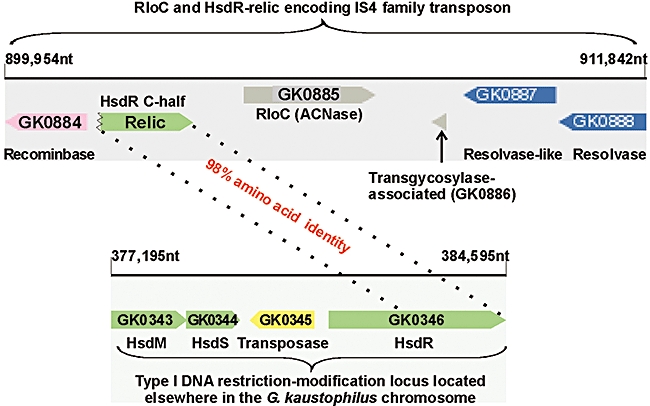
*G. kaustophilus rloC* is linked to an *hsdR* relic. The schemes describing the region about *G. kaustophilus rloC* and its flanking *hsdR* relic and the complete *hsd* locus located elsewhere in the *G. kaustophilus* genome were adapted from the annotated genomic NCBI map (Takami *et al*., 2004). Numbers followed by nt indicate positions in the genomic map. The non-coding region separating locus tags GK0884-5 was found by blast (Altschul *et al*., 1997) to encode a C-terminal 568 aa portion of an HsdR species that was 98% identical in amino acid sequence with the matching portion of the *hsdR* gene of the complete locus (GK0346).

As mentioned, known zinc-hook/coiled-coil proteins other than RloC partake in DNA repair and related transactions (Hopfner and Tainer, 2003). This fact and the implied regulatory role of RloC's zinc hook raise the possibility that RloC ACNase is mobilized by DNA damage. However, other observations suggest that RloC can be regulated by the state of an associated DNA restriction nuclease. Namely, a few RloC orthologues resemble PrrC in being linked to a type I or the related type III R-M locus (Table 1, Table S1, Miller *et al.*, 2005). Moreover, many of the remaining orthologues exist in bacteria encoding these R-M systems and, hence, could in theory interact with them *in trans*. The latter assumption is reinforced by the following observations. First, *G. kaustophilus rloC* resides in an IS4 family transposon flanked upstream by the cryptic C-half of a type I restriction subunit gene (*hsdR*) (Fig. 7). This relic is ∼98% identical with the matching portion of a complete *hsd* locus located elsewhere in the *G. kaustophilus* genome but less similar to other *hsdR* genes of closely related bacteria. These coincidences suggest that the flanking *hsdR* relic stemmed from a full-fledged R-M system that interacted with RloC *in cis* but was superseded by a duplicate able to exert this function *in trans*. Second, two RloC orthologues are linked to the anti-DNA restriction factor ArdC (Belogurov *et al*., 2000), hinting they are activated when an associated DNA restriction enzyme is compromised. One of these ArdC-linked RloC orthologues is also linked to a type III R-M system. The other could in theory interact with a type I R-M system but only *in trans*. As for the RloC orthologues of bacteria lacking a suitable R-M system, at least some of them could be inactive, as inferred from their poor ATPase or ACNase motifs.

Accordingly, RloC could respond both to DNA damage-related cues with its Rad50-like domain and, in ‘PrrC's way', to the state of an associated DNA restriction nuclease. It may be further asked if such putative controls cooperate, function independently or act in a mutually exclusive manner. We favour the first possibility because DNA damage leads to alleviation of type I DNA restriction (Thoms and Wackernagel, 1984) through proteolysis of the DNA restriction subunit of the type Ia system or unidentified alteration of the type Ic system (Thoms and Wackernagel, 1984; Makovets *et al*., 1999; Blakely and Murray, 2006). This precautionary measure prevents degradation of fully unmodified cellular DNA synthesized by homologous recombination during the recovery from DNA damage. However, at the same time, it renders the cell vulnerable to phages that escape DNA restriction (Blakely and Murray, 2006). RloC could benefit its host cell population in such a situation, responding to DNA damage and/or consequent inactivation of the associated DNA restriction nuclease by disabling translation, and thus ward off phages that escape DNA restriction during the recovery. We cannot exclude that RloC is mobilized by DNA damage also irrespective of phage infection. However, such activation is expected to inhibit the synthesis of DNA repair and other stress-related proteins normally induced in bacteria exposed to genotoxic stress. Therefore, RloC would function also in that case as a suicidal device that benefits unaffected members of the cell population.

### The RloC lesion could defy reversal by phage tRNA repair enzymes

The cleavage of tRNA^Lys^ by PrrC is counteracted by tRNA repair-competent phage (Amitsur *et al*., 1987; 2003; Miller *et al*., 2003; Blondal *et al*., 2005). However, the lesion inflicted by RloC, excision of the wobble nucleotide, could defy such reversal and render RloC a more potent antiviral device than PrrC. Such advantage could account for the approximately threefold higher incidence of RloC among bacteria. It may be argued against this assumption that the phage 3′-healing function (CPD) can pre-empt the excision of the wobble nucleotide by dephosphorylating the initial incision product (Fig. 4). However, *in vitro* only a minor fraction of the intermediate was accessed by CPD, the occluded bulk being further processed and the wobble nucleotide trimmed from it. Whether RloC excises the wobble nucleotide in the presence of phage tRNA repair enzymes also *in vivo* remains an open question. This uncertainty and the need to examine other assumptions made here about RloC call for studying this ACNase in situations closer to the physiological, including perhaps encounters with tRNA repair competent phage.

## Experimental procedures

### Materials

DNA oligonucleotides were purchased from Life Technologies and Sigma-Genosys, T4 polynucleotide kinase from USB Biochemicals, T4 RNA ligase and DNA restriction nucleases from New England Biolabs, RNases T1 and T2 from Sigma, Nuclease P1 and anti-His_6_ mouse monoclonal antibody from Roche Applied Science, *Pfu* DNA polymerase from Stratagene and [γ-^32^P]-ATP from Amersham.

### Cloning, expression and isolation of RloC

The *G. kaustophilus* HTA426 gene encoding an 804 aa RloC protein (NCBI accession YP_146738) was amplified by PCR from genomic DNA using *Pfu* DNA polymerase. The PCR primers introduced an NdeI restriction site at the start codon and an AgeI site at the C-end to fuse it via a flexible linker to a His_6_ tag, as in the PrrC plasmid pRRC11-L-His_6_ (Blanga *et al*., 2006). The PCR product digested with NdeI and AgeI restriction nucleases replaced the PrrC portion of pRRC11-L-His_6_ to yield pGkaRloC-L-His_6_. Amino acid replacements were performed by Quick Change (Ansaldi *et al*., 1996). pGkaRloC-L-His_6_ and its mutant derivatives were transformed into *E. coli* DH10B and, after confirming their sequence, into *E. coli* Rosetta™ (DE3)pLysS (Novagen). The transformants were grown to 0.4 OD_600_ at 37°C in Luria–Bertani (LB) medium containing 100 μg ml^−1^ ampicilin and 34 μg ml^−1^ chloramphenicol. Isopropyl-β-d-thiogalactopyranoside (IPTG) was added at 0.1 mM to induce expression. The culture was shifted to 30°C and incubated for 20 min. All subsequent steps were at 0–4°C. The cells were harvested by centrifugation and the pellet was washed twice in buffer I [10 mM Tris–HCl (pH 7.5); 15 mM MgCl_2_, 1 M KCl and 10% glycerol], once in buffer II (buffer I with 50 mM KCl). Aliquots were withdrawn for detection of the expressed protein by immunoblotting and isolating total low-weight RNA for detection of ACNase cleavage products (Meidler *et al*., 1999). The bulk of the cells were suspended at 1:1.5 w/v in buffer III [50 mM Tris-HCl, pH 7.5; 10 mM MgCl_2_, 1 mM DTT, Protease inhibitor cocktail tablet, EDTA-free (Roche); and 10% glycerol]. The cells were disrupted in an Amicon pressure cell at 18 000 psi and the lysate was centrifuged for 30 min at 30 000 *g* in a Sorvall SS-34 rotor. The supernatant containing ∼30 mg ml^−1^ protein (30S fraction) was stored at −20°C or fractionated by immobilized cobalt affinity chromatography essentially as described for PrrC (Blanga-Kanfi *et al*., 2006). Briefly, the S30 fraction total protein was supplemented with 5 mM imidazole and 2 ml of it loaded on a 0.5 ml or 6 ml TALON column equilibrated with buffer RI (50 mM Tris-HCL pH 7.5; 10 mM MgCl_2_, 1 mM DTT and 5 mM imidazole). The column was washed with 10 volumes of the same buffer (RI) and the bound protein eluted with buffer RIII (buffer R with 0.5 M imidazole). The RloC fractions were monitored by their ACNase activity, immunoblotting and, where indicated, protein staining.

### ACNase assays

A previous protocol was used to determine ACNase activity *in vivo* by 5′-end-labelling the resulting 3′-RNA *in vitro* using T4 Pnk (Meidler *et al*., 1999). The 5′-end-labelled *in vivo* cleavage products were also further treated with T4 RNA ligase to restore the tRNA substrates by supplementing aliquots of the labelling mixture at the end of polynucleotide kinase reaction with 2 mM ATP and 1 unit μl^−1^ T4 RNA ligase 1. The mixture was further incubated for 1 h at 37°C and the products were separated by denaturing gel electrophoresis. The *in vivo* cleavage products of PrrC or RloC and their ligated derivatives were also visualized by ethidium bromide staining.

ACNase was assayed *in vitro* using tRNA^Lys^ radiolabelled at the 33p34 junction as a substrate and S30 extracts of cells expressing the indicated RloC form or PrrC-D222E, essentially as described (Amitsur *et al*., 1989; Blanga-Kanfi *et al*., 2006), except that the RloC-containing reaction mixtures (10 μl) were adjusted to 50 mM Tris-HCl, 1 mM DTT and 10 mM MgCl_2_. They contained 10–15 μg of S30 fraction total protein or a corresponding volume of the IMAC-purified fraction. Where indicated, these reaction mixtures were supplemented with 30 units of T4 polynucleotide kinase (USB).

### Analyses of ACNase cleavage products

PrrC's or RloC's substrate preference was assessed by hybridizing their 5′-labelled *in vivo* cleavage products to antisense DNA oligonucleotides complementary to 3′ portions of the indicated tRNAs. Some of these probes have been previously described (Jiang *et al*., 2001) and others are listed in Table S2. Enzymatic treatments of the 5′-end-labelled fragments and the derived, internally labelled ligation products with RNase T1, RNase T2, nuclease P1 or the CPD activity of T4 Pnk were performed essentially as previously described (Amitsur *et al*., 1987; 1989) and the labelled nucleotides released were separated by a 2D TLC protocol (Nishimura, 1979)
