# Supplementary material for: RloC: a wobble nucleotide-excising and zinc-responsive bacterial tRNase
Source: Mol Microbiol. 2008 Aug 7;69(6):1560–74. doi: 10.1111/j.1365-2958.2008.06387.x (PMC2610378; doi:10.1111/j.1365-2958.2008.06387.x)
Supplement: Supplementary file 1 [file mmi0069-1560-SD1.pdf]

**Supplementary Table 1. Properties of RloC orthologs**

| Domain/ Division/ Organism | Accession | aa | Hsd[a] | Flanking genes | Other features of interest |
|---|---|---|---|---|---|
| ***Archaea*** | | | | | |
| 1. Methanocorpusculum labreanum Z | YP_001029478 | 743 | + | Hypothetical | |
| ***Bacteria*** **Firmicutes** | | | | | |
| 2. Geobacillus kaustophilus HTA426 | YP_146738 | 804 | + | Transposase, C-half of R subunit of Type I R-M sys., Resolvase | |
| 3. Petrotoga mobilis SJ95 | ZP_01777264 | 757 | + | Type IIS restriction system | |
| 4. Staph. aureus subsp. aureus MRSA252 | YP_039547 | 706 | + | Protein kinase, Transposase | |
| 5. Listeria monocytogenes str. 4b F2365 | YP_013291 | 712 | - | ST phosphatase | |
| 6. Fusobacterium nucleatum subsp. vincentii ATCC | EAA23309 | 702 | + | ATP dependent nuclease | Encodes PnkP |
| 7. Clostridium thermocellum ATCC 27405 | YP_001037586 | 800 | + | S23 ribosomal protein; Glc--Frc-6-P aminotransferase | Encodes PnkP |
| 8. Lactobacillus delbrueckii subsp. bulgaricus | YP_813123 | 736 | + | Possible cation transport ATPase | |
| 9. Thermosipho melanesiensis BI429 | EAX35558 | 741 | + | Metallo-phosphoesterase, Short hydrophilic protein | Cryptic P-loop (GX4GKF) |
| **Actinobacteria** | | | | | |
| 10. Mycobacterium sp. MCS | YP_639760 | 887 | + | phage integrase | |
| 11. Mycobacterium sp. KMS | YP_938625 | 887 | + | Integrase, pseudogene | |
| 12. Brevibacterium linens BL2 | ZP_00378003 | 855 | + | Ade deaminase. Excinuclease. ATPase | Encodes also PrrC |
| 13. Corynebacterium diphtheriae NCTC 13129 | NP_939996 | 746 | + | Hypothetical | |
| **Bacterioidetes** | | | | | |
| 14. Chlorobium tepidum TLS | NP_661579 | 767 | + | Cryptic type I R-M | |
| 15. Chlorobium phaeobacteroides BS1 | EAM64071 | 743 | + | Hypothetical | |
| 16. Chlorobium limicola DSM 245 | EAM42052 | 869 | + | Type I Methylase; Type III restriction | |
| 17. Chlorobium chlorochromatii CaD3 | YP_380258 | 844 | + | Thiosulfate reductase, Fe-S-cluster hydrogenase, Glutamate-1- | |
| 18. Leeuwenhoekiella blandensis MED217 | ZP_01061349 | 715 | + | Type IV Eco57I related | |
| 19. Pedobacter sp. BAL39 | ZP_01883553 | 849 | + | Hypothetical | |
| 20. Psychroflexus torquis ATCC 700755 | ZP_01253815 | 873 | + | Putative transposase, resolvase | |
| **Proteobacteria (subdivision)** | | | | | |
| 21. Mariprofundus ferrooxydans PV-1 (others) | ZP_01451087 | 688 | + | Hypothetical | |
| 22. Bradyrhizobium sp. BTAi1 - α-subdiv. | YP_001243161 | 874 | + | DNA repair RadC, integrase, ArdC | Encodes PnkP |
| 23. Hyphomonas neptunium ATCC15444 (α). | YP_759015 | 747 | + | DNA binding domain | |
| 24. Labrenzia aggregata IAM 12614 (α) | EAV41045 | 763 | - | DNA repair exonuclease, AraC family regulator | |
| 25. Nitrobacter hamburgensis X14 (α) | YP_579004 | 895 | - | Cell division, DNA methylase, Resolvase | Suboptimal ACNase triad |
| 26. Nitrobacter winogradskyi Nb-255 (α) | YP_316886 | 872 | + | Type I R-M , Phage integrase | |
| 27. Rhodobacterales bacterium HTCC2150 (α) | ZP_01740288 | 870 | + | Type III R-M, ArdC | |
| 28. Rhodopseudomonas palustris BisB18 (α) | YP_534072 | 722 | + | Sigma 24 (FecI-like), Adenosine deaminase | |
| 29. Roseovarius nubinhibens ISM (α) | ZP_00959683 | 798 | - | Hypothetical | |
| 30. Sphingomonas wittichii RW1 (α) | YP_001260375 | 881 | - | Carbamoyl transferase | Encodes type III R-M system |
| 31. Burkholderia phymatum STM815 (β) | ZP_01505650 | 884 | - | DNA ligase, diguanylate cyclase, Helicase-like | Poor ACNase triad? |
| 32. Burkholderia pseudomallei K96243(β) | YP_109854 | 806 | + | Plasmid recombinase, plasmid conjugal transfer | |

| | | | | | |
|---|---|---|---|---|---|
| 33. Burkholderia pseudomallei 305 (β) | ZP_01767292 | 733 | - | Hypothetical | |
| 34. Burkholderia sp. 383 (β) | YP_369178 | 878 | - | Hypothetical | |
| 35. Burkholderia xenovorans LB40 (β) | YP_556597 | 833 | + | DNA replication (DnaA, DnaN, GyrB); Transposase | Poor ACNase triad? |
| 36. Ralstonia solanacearum GMI1000 -(β) | NP_520740 | 767 | + | Probable homolog of g32 F-plasmid | |
| 37. Ralstonia metallidurans CH34 (β) | YP_587691 | 792 | - | Transcriptional regulator | |
| 38. Acidovorax sp. JS42 (β) | YP_986869 | 767 | + | DNA repair RadC | |
| 39. Rhodoferax ferrireducens T118 (β) | YP_515976 | 770 | + | Helicase like | |
| 40. Pelobacter propionicus DSM 2379e (δ) | YP_899804 | 869 | + | Transposase, pseudogene | |
| 41. Delta proteobacterium MLMS-1 (δ) | EAT05858 | 683 | + | Type I R-M | |
| 42. Syntrophobacter fumaroxidans MPOB (δ) | YP_846144 | 897 | + | Transposase | |
| 43. Campylobacter jejuni subsp. jejuni 260.94 (ε) | ZP_01069526 | 714 | + | Hypothetical | |
| 44. Campylobacter jejuni subsp. jejuni 84-25 (ε) | EAQ93916 | 714 | + | Type II R-M; putative phage terminase | |
| 45. Campylobacter jejuni RM1221 (ε) | YP_179697 | 776 | + | Cryptic Type I R-M | |
| 46. Helicobacter pylori HPAG1 (ε) | YP_627821 | 815 | + | Fmet-tRNA formyl transferase | |
| 47. Helicobacter pylori 26695 (ε) | NP_207933; | 759 | + | Fmet-tRNA formyl transferase | |
| 48. Helicobacter pylori J99 (ε) | NP_223787 | 759 | + | Fmet-tRNA formyl transferase | |
| 49. Helicobacter acinonychis str. Sheeba (ε) | YP_664387 | 811 | + | Hypothetical | |
| 50. Erwinia carotovora subsp. atroseptica SCRI1043 (γ) | YP_049069 | 868 | + | Putative acetyl transferase, TA antitoxin; Helix-turn-helix putative | |
| 51. Escherichia coli APEC O1 (γ) | YP_852004 | 738 | + | Phage replication initiation, Capsid | Encodes type I EcoK like |
| 52. Yersinia pseudotuberculosis IP31758 (γ) | ZP_01498189 | 728 | + | Hypothetical | |
| 53. Acinetobacter sp. ADP1 (γ) | YP_044948 | 826 | + | GMP synthase, Toxin-antitoxin | |
| 54. Idiomarina loihiensis L2TR (γ) | YP_156470 | 863 | + | α/β family hydrolase | |
| 55. Marinobacter aquaeolei VT8 (γ) | YP_957854 | 810 | + | SbcCD | |
| 56. Pseudoalteromonas tunicata D2 (γ) | ZP_01131820 | 758 | + | Hypothetical, reverse transcriptase | |
| 57. Shewanella putrefaciens CN-32 (γ) | YP_001185489 | 803 | + | Type I R-M | |
| 58. Shewanella sp. MR-4 (γ) | YP_732608 | 771 | + | DNA helicase II | |
| 59. Shewanella baltica OS195 - γ-subdiv | EAU25632 | 771 | + | DEAD/DEAH box helicase-like | |
| 60. Shewanella sp. ANA-3 plasmid 1 - γ-subdiv | YP_863754 | 746 | + | Transposase, IS605 OrfB-like, IS200-like transposase | |
| 61. Shewanella sp. W3-18-1 - γ-subdiv | YP_962600 | 861 | + | Metallophosphoesterase, Protein-nucleoside recognition domain | |
| 62. Pseudomonas aeruginosa UCBPP-PA14 (γ)<br>63. Pseudomonas aeruginosa UCBPP-PA14 (γ) | YP_789389<br>YP_791302 | 868<br>717 | -<br>- | Possible dehydrogenase<br>Hypothetical | poor ACNase triad,<br>poor Walker B |
| 64. Pseudomonas putida KT2440 (γ) | NP_745825 | 891 | + | DNA helicase related; Toxin-antitoxin | |
| 65. Pseudomonas fluorescens PfO-1 (γ) | YP_348159 | 849 | - | Hypothetical | Poor ACNase triad? |
| 66. Pseudomonas syringae pv.syringae B728a (γ) | YP_235721 | 643 | - | Hypothetical | Missing Walker A |
| 67. Pseudomonas aeruginosa PA7 (γ) | ZP_01296427 | 797 | + | Hypothetical | |
| 68. Vibrio parahaemolyticus RIMD 2210633 (γ) | NP_800765 | 771 | + | Transposase | |
| 69. Vibrio splendidus 12B01 (γ) | ZP_00991284 | 850 | + | Hypothetical | Encodes also PrrC |
| 70. Vibrio alginolyticus 12G01 (γ) | ZP_01259550 | 866 | + | Hypothetical | |
| 71. Xanthomonas oryzae pv. Oryzae (N-truncated) (γ) | YP_198670 | 658 | + | Putative Transposase, Transposase | |
| 72. Xylella fastidiosa 9a5c (γ) | NP_299690 | 777 | + | Hypothetical | |

The indicated RloC orthologs were retrieved by repeated homology searches in NCBI's microbial genome database using BLAST (Altschul *et al.*, 1997). *C. jejuni* RM1221 RloC was used as the initial query and this was followed by using various distantly related orthologs and subsequent queries. All candidates were screened for presences of ABC ATPase, CXXC and putative ACNase triad motifs. Several possible orthologs seemingly truncated, either due to a deletion or misinterpreted sequence analysis are not included in the table. a. presence (+) or absence (-) of a type I or type III DNA R-M system in the indicated organism.

## REFERENCES

1. Altschul SF, Madden TL, Schaffer AA, Zhang J, Zhang Z, Miller W, and Lipman DJ (1997) Gapped BLAST and PSI-BLAST: a new generation of protein database search programs. *Nucleic Acids Res,* **25,** 3389-3402.

## Supplementary Table 2 - Expected ACNase sites in T1 anticodon region oligonucleotides

| | PrrC | | | RloC | | |
|---|---|---|---|---|---|---|
| **tRNA** | Cleavage site within T1 fragment | a | b | Cleavage sites within T1 fragment | c | d |
| **Lys** | ACUCU↓mnm$^5$s$^2$UUUt$^6$AAΨCAAUUG | 17 | 12 | CUCU↓mnm$^5$s$^2$U↓UUt$^6$AAΨCAAUUG | 16 | 11 |
| **Asn** | ACU↓QUUt$^6$AAΨCCG | 12 | 9 | ACU↓Q↓UUt$^6$AAΨCCG | 11 | 8 |
| **Glu** | CCCU↓mnm$^5$s$^2$UUCm$^2$ACG | 10 | 6 | CCCU↓mnm$^5$s$^2$U↓UCm$^2$ACG | 9 | 5 |
| **Gln1** | AUmU↓CUG | 6 | 3 | AUmU↓C↓UG | 5 | 2 |
| **Gln2** | AUm U↓mnm$^5$s$^2$UUG | 6 | 3 | AUmU↓mnm$^5$s$^2$U↓UG | 5 | 2 |
| **His** | AUU↓QUG | 6 | 3 | AUU↓Q↓UG | 5 | 2 |
| **Arg1,2** | s$^2$CU↓I; CU↓I; | 3 | 1 | s$^2$CU↓I↓CG; CU↓I↓CG | 4 | 2 |
| **Arg3** | CCCs$^2$CU↓mnm$^5$s$^2$UCUt$^6$AAG | 11 | 6 | CCCs$^2$CU↓mnm$^5$s$^2$U↓CUt$^6$AAG | 10 | 5 |
| **Arg4** | ACs$^2$CU↓mnm$^5$s$^2$UCUt$^6$AAG | 10 | 6 | ACs$^2$CU↓mnm$^5$s$^2$U↓CUt$^6$AAG | 9 | 5 |
| **Arg5** | CCs$^2$CU↓CCG | 7 | 3 | CCs$^2$CU↓C↓CG | 6 | 2 |
| **Asp** | CCU↓QUCm$^2$ACG | 9 | 6 | CCU↓Q↓UCm$^2$ACG | 8 | 5 |
| **Ala** | CAU↓G<br>CUU↓$^{cmo5}$UG | 4<br>5 | 1<br>2 | CAUG↓G↓CAUG<br>CUU↓cmo$^5$U↓G | 4<br>4 | 4<br>1 |
| **Cys** | AΨU↓G | 4 | 1 | AΨU↓G↓CAms$^2$AAΨCCG | 11 | 8 |
| **Phe** | AΨU↓G | 4 | 1 | AΨU↓G↓AAms$^2$AAΨCCCCG | 13 | 10 |
| **Gly** | CUU↓CCCAAG<br>ACCUU↓G<br>CUU↓$^{unk}$UCCAAG | 9<br>6<br>9 | 6<br>1<br>6 | CUU↓C↓CCAAG<br>ACCUU↓G↓CCAAG<br>CUU↓unkU↓CCAAG | 8<br>10<br>8 | 5<br>4<br>5 |
| **Ile** | CACCCCU↓G<br>ACU↓$^{k2}$CAUt$^6$AΨCG | 8<br>10 | 1<br>7 | CACCCCU↓G↓AUt$^6$AAG<br>ACU↓k$^2$C↓AUt$^6$AΨCG | 12<br>9 | 5<br>6 |
| **Leu** | ΨU↓unkAAAmS$^2$i$^6$AAΨCCCUCG<br>CUU↓CAG | 14<br>6 | 12<br>3 | ΨU↓unkA↓AAmS$^2$i$^6$AAΨCCCUCG<br>CUU↓C↓AG | 13<br>5 | 11<br>2 |
| **Met** | CACAUCACU↓ac4CAUt6AAΨG | 16 | 7 | CACAUCACU↓ac$^4$C↓AUt6AAΨG | 15 | 6 |
| **Pro** | UmU↓CG<br>UCCmU↓G<br>UmU↓cmo$^5$UG | 4<br>5<br>4 | 2<br>1<br>2 | UmU↓C↓G<br>UCCmU↓G↓G<br>UmU↓cmo$^5$U↓G | 3<br>4<br>3 | 1<br>1<br>1 |
| **Ser** | UCU↓CG<br>CUCCCs$^2$CU↓G<br>CCU↓G<br>UCmU↓cmo$^5$UG | 5<br>8<br>4<br>5 | 2<br>1<br>1<br>2 | UCU↓C↓G<br>CUCCCs$^2$CU↓G↓CUt$^6$AAG<br>CCU↓G↓G<br>UCmU↓cmo$^5$U↓G | 4<br>12<br>4<br>4 | 1<br>5<br>1<br>1 |
| **Thr** | CACCCUU↓G | 8 | 1 | CACCCUU↓G↓G | 8 | 1 |
| **Val** | CACCACCUU↓G<br>CACCUCCCU↓cmo$^5$UACm$^6$AAG | 10<br>15 | 1<br>6 | CACCACCUU↓G↓ACAUG<br>CACCUCCCU↓cmo$^5$U↓ACm$^6$AAG | 14<br>14 | 5<br>5 |
| **Trp** | UCmU↓CCAms$^2$i$^6$AAACCG | 12 | 9 | UCmU↓C↓CAms$^2$i$^6$AAACCG | 11 | 8 |
| **Tyr** | ACU↓QUAms$^2$i$^6$AAΨCUG | 12 | 9 | ACU↓Q↓UAms$^2$i$^6$AAΨCUG | 11 | 8 |
| **fMet** | CmU↓CAUAACCCG | 11 | 9 | CmU↓C↓AUAACCCG | 10 | 8 |
| **SelC** | ACU↓UCAi$^6$AAUCCAG | 12 | 9 | ACU↓U↓CAi$^6$AAUCCAG | 11 | 8 |

a – Size of labeled T1-oligonucleotides derived from religated PrrC products.
b - Size of labeled T1-oligonucleotides derived from 3' cleavage product of PrrC
c – Size of labeled T1-oligonucleotides derived from the religated RloC products, assuming excision of the wobble nucleotide.
d - Size of labeled T1-oligonucleotides derived from 3' cleavage product of RloC.
↓ indicates expected cleavage site(s) of the respective ACNase.
It should be pointed out that certain base modifications confer positive (mnm$^5$s$^2$U) or negative charge (cmo$^5$U) and, therefore, reduce or enhance in respective order the mobility of the oligonucleotides containing them below or above that expected from their size

**Supplementary Table 3**. New DNA oligonucleotides probes used to identify tRNA species cleaved by PrrC and RloC.

| tRNA species | Antisense DNA oligonucleotide | tRNA residues it corresponds to |
|---|---|---|
| Ala[a] | TGGAGCTATGCGGGATCGAACCGCAGACCTC | 43-73 |
| Arg1,2 | TGCATCCGGGAGGATTCGAACCTCCGACCGCTCGGT | 38-73 |
| Arg3 | TGTCCCCTGCAGGAATCGAACCTGCAATTAGCCCT | 38-73 |
| Arg4 | CGCGCCCTGCAGGATTCGAACCTGCGGCCCACGACT | 38-73 |
| Cys | GGCGCGTTCCGGAGTCGAACCGGACTAGACGG | 40-72 |
| Gly[b] | AGCGGGCAGCGGGAATCGAACCCGCATC | 45-73 |
| His | GGTGGCTAATGGGATTCGAACCCACGACAACTGGAA | 38-73 |
| Pro[c] | TCGGCGAGAGAGGATTCGAACCTCCGACCC | 44-73 |
| Thr[d] | TGCTGATACCCAGAGTCGAACTGGGGACC | 45-73 |
| Trp | CGGAGAGACTCGAACTCCCAACACCC | 42-67 |

[a]- Complementing $tRNA^{Ala1\&3}$ but expected to also cross-hybridize $tRNA^{Ala2}$
[b] – Complementing $tRNA^{Gly3}$ but expected to also cross-hybridize $tRNA^{Gly1\&2}$
[c]- Complementing $tRNA^{Pro}$ gene 1 product but could cross-hybridize those of genes 2 & 3
[d] - Complementing $tRNA^{Thr}$ gene 5 product but could cross-hybridize those of genes 1 & 3

**Supplementary Figure 1**

**A**

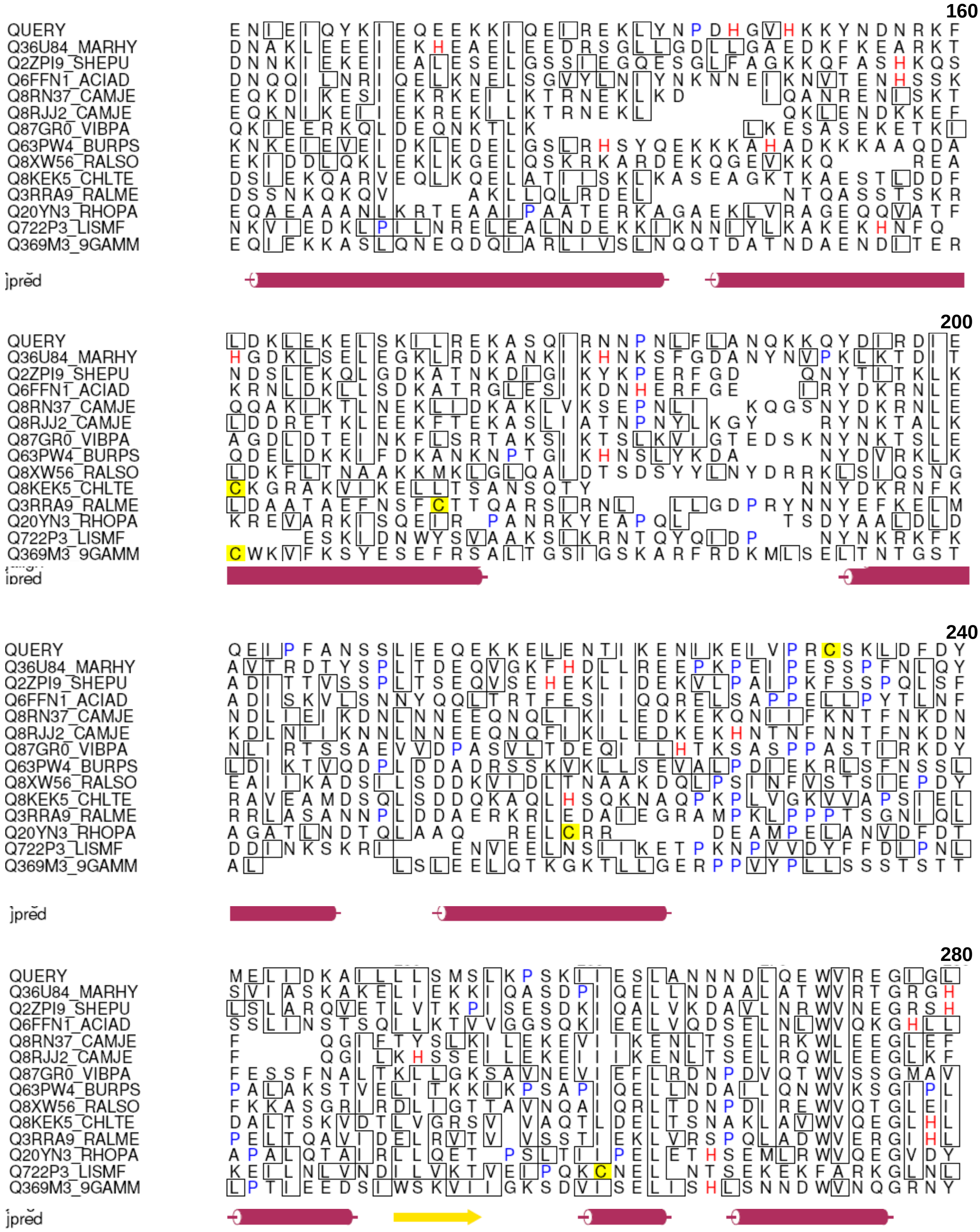

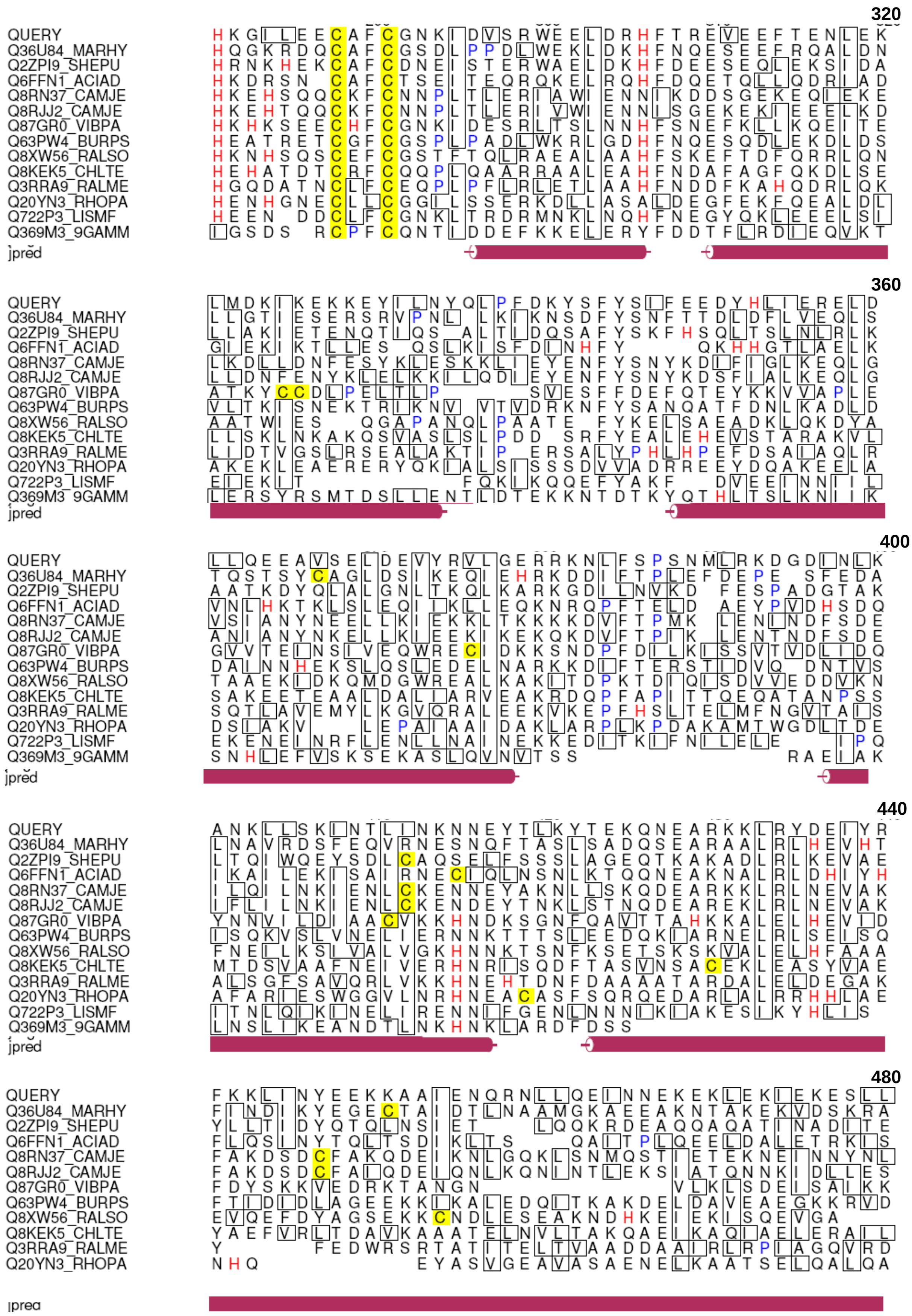
320
360
400
440
480
QUERY
Q36U84_MARHY
Q2ZPI9_SHEPU
Q6FFN1_ACIAD
Q8RN37_CAMJE
Q8RJJ2_CAMJE
Q87GR0_VIBPA
Q63PW4_BURPS
Q8XW56_RALSO
Q8KEK5_CHLTE
Q3RRA9_RALME
Q20YN3_RHOPA
Q722P3_LISMF
Q369M3_9GAMM
jpred

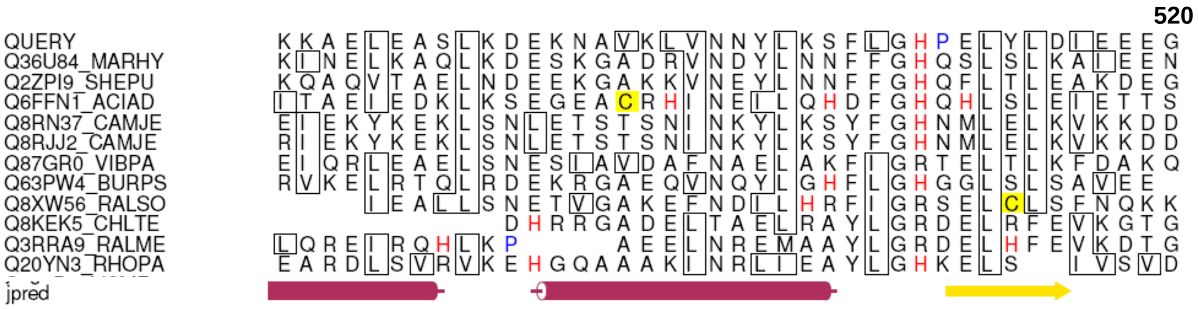


**Legend.** The secondary structure of *G. kaustophilus* RloC was predicted by Jpred (Cuff *et al.*, 1998). The portion of the query shown spans residues 121-540 and contains the zinc hook motif CXXC whose two cysteines are highlighted and the flanking, largely α-helical regions.

Reference

Altschul,S.F., Madden,T.L., Schaffer,A.A., Zhang,J., Zhang,Z., Miller,W., and Lipman,D.J. (1997) Gapped BLAST and PSI-BLAST: a new generation of protein database search programs. *Nucleic Acids Res* **25**: 3389-3402.

**Supplementary Figure 2**

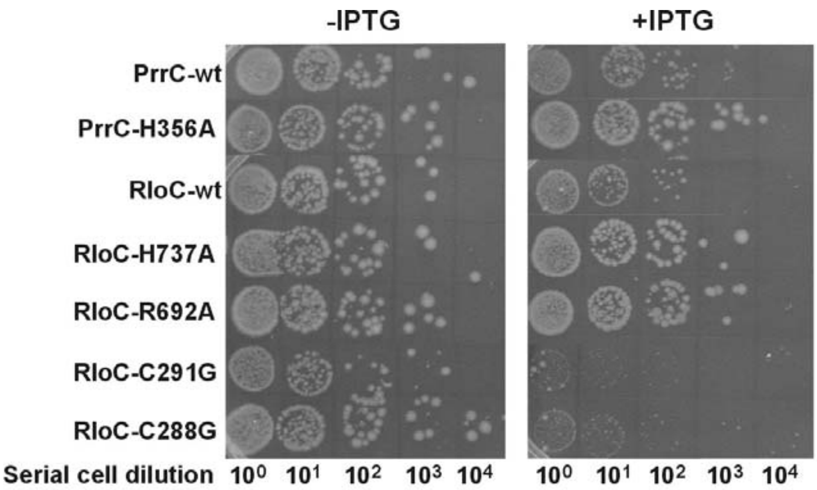


**Legend. Toxicity assay of PrrC, RloC and mutant derivatives.** *E. coli* Rosetta cells transformed with the indicated PrrC or RloC clones were plated at the indicated serial dilutions on growth plates without or with 4μM IPTG.

**Supplementary Figure 3**

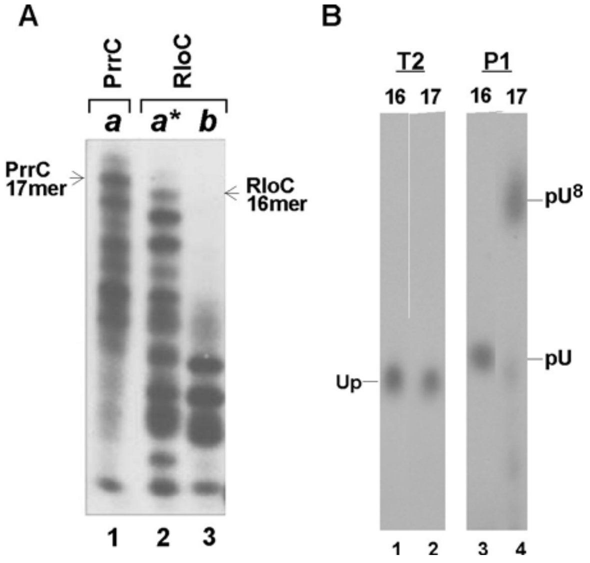


**Legend. Nucleotides flanking the ligation junction in ligated back cleavage products of tRNA$^{Lys}$ generated by PrrC or by RloC.** A. T1 oligonucleotide pattern of the religated PrrC or RloC cleavage products. The religated back molecules from the indicated bands from Fig. 3A lanes 7 and 8 were exhaustively digested with RNase T1 and the labeled oligonucleotides containing the ligation junctions separated by denaturing gel electrophoresis. The largest oligomers in the PrrC set is a 17mer derived from tRNA$^{Lys}$ and that of the RloC set a 16 that could also be derived only from tRNA$^{Lys}$ (other tRNA species would have yielded smaller T1 oligonucleotides containing the cleavage junction, Supplementary Table 2). B. The PrrC 17mer and RloC 16mer were digested with RNase T2 or nuclease P1. The first treatment released labeled Up from either oligonucleotide. The second treatment released the wobble nucleotide pU$^8$ from PrrC's 17mer and pU from RloC's 16mer. These results indicated that the wobble nucleotide was excised from tRNA$^{Lys}$ in the RloC-expressing cells. None of six other T1 oligonucleotides derived from the ligated-back RloC cleavage products of bands *a** and *b* and similarly analyzed contained a wobble nucleotide at the ligation junction (not shown).
